# Exploring the Factors behind Nurses’ Decision to Leave Clinical Practice: Revealing Causes for Leaving and Approaches for Enhanced Retention

**DOI:** 10.3390/healthcare11243104

**Published:** 2023-12-05

**Authors:** Raeed Alanazi, Ghareeb Bahari, Zahra Ali Alzahrani, Abdulelah Alhaidary, Kholoud Alharbi, Bander Saad Albagawi, Naif H. Alanazi

**Affiliations:** 1 Nursing Administration and Education Department, College of Nursing, King Saud University, Riyadh 11421, Saudi Arabia; raalenazi@ksu.edu.sa (R.A.); kalharbi1@ksu.edu.sa (K.A.); 2 Nursing Administration, King Faisal Hospital-Makkah, Makkah 24236, Saudi Arabia; alzahranizahra255@gmail.com; 3 Nursing Administration, King Saud University Medical City, Riyadh 11472, Saudi Arabia; aalhaidary1@ksu.edu.sa; 4 Medical Surgical Department, College of Nursing, University of Hail, Hail City 2440, Saudi Arabia; saad1510@hotmail.com; 5 Medical Surgical Department, College of Nursing, King Saud University, Riyadh 11421, Saudi Arabia

**Keywords:** nurses, nursing profession, intention to leave work, nursing turnover

## Abstract

Nursing turnover has emerged as an urgent concern with a substantial influence on the financial efficiency and quality of care in healthcare frameworks worldwide. This study determined important factors associated with nurses’ intentions to leave and what would bring them back. This was a cross-sectional, multisite study of nurses in three public hospitals. Convenience sampling was used to recruit 205 nurses from the selected hospitals. A questionnaire was used to measure demographic and professional background information, current job satisfaction, and the intention to leave work and return. Bivariate and multivariate analyses were run using SPSS. This study revealed that both job satisfaction (M = 47.26, SD = 11.59, range: 19–76) and intent to leave a current job (M = 14.18, SD = 4.36, range: 4–20) were reported at moderate levels. There were significant differences reported between the scores of nationality and job satisfaction (*p* < 0.05) and between the means of income level and intention to leave (*p* < 0.05). There was also a significant, negative association between satisfaction and intention to leave (r = −0.551, *p* < 0.05). In regression, income level (β = 0.159, *p* = 0.021), incentives (β = 0.186, *p* = 0.002), hospital type (β = 0.189, *p* = 0.005), and intention to leave (β = −0.454, *p* < 0.001) significantly influenced satisfaction. Gender (β = −0.122, *p* = 0.037) and nationality (β = −0.210, *p* = 0.007) were found to influence the intention to leave among participants significantly. In conclusion, this study indicated that job satisfaction and intention to leave are important factors affecting nurses’ enthusiasm. Incentives also had a positive impact on increasing nurses’ satisfaction levels. Future research studies should investigate what factors might lead to improved monthly salaries and provide more incentives among nurses.

## 1. Introduction

The Kingdom of Saudi Arabia’s health system is experiencing change as part of the National Transformation Program, which is essential for Saudi Arabia’s long-term desire, Vision 2030 [1]. The difficulties could include an absence of nursing school limits and a higher opportunity to ostracize business [2]. The limitations of nursing schools across the country can pose a significant problem in graduating enough qualified nurses to meet healthcare needs. Strategic planning and investment in nursing are also necessary to address issues such as lack of support and limited opportunities for professional growth. Creating an environment that supports excellence would enhance effective cooperation among nurses and reduce feelings of marginalization or ostracism, if available, ultimately promoting longer-term retention in the profession. Nurses’ plans to leave their current profession have turned into a global matter studied broadly over the previous decade [2]. It has been legitimated as a voluntary and early termination of nursing staff [3]. Regardless, the intention to leave has been extensively portrayed as an individual’s expectation of quitting their current employer sooner rather than later. Insufficient nursing staff, expanding work stress attributable to rising workloads, job discontent, and the aim to leave and move to other healthcare organizations are the most genuine hardships that healthcare organizations face [4].

The turnover rate of registered nurses is a key sign of the nurse labor market. Although turnover is frequently used as a metric for analyzing the healthcare system, its definition is inconsistent. Some authors define turnover as any nurse leaving the healthcare organization, while others define it as the involuntary and voluntary leaving of nurses [5]. Nurse turnover is a human resources issue that influences healthcare globally. The rate ranges from 15 to 44% and is relatively high [6]. Nurse turnover in healthcare institutions can significantly impact patient care and lower the standard of treatment. Additionally, a rising nurse turnover rate in hospitals results in a lack of personnel, increasing the burden and stress on other nurses. Adjustments and inadequate staffing brought on by nurse turnover can lead to increases in infection and death rates, medication errors, and treatment mistakes [7].

Expanded turnover is likewise unsafe for patient health [8]. According to a new report, higher nurse turnover prompts lengthier hospitalization and a greater frequency of medical mistakes [9]. However, the need to replace employees and enlist and train temporary staff brings about higher costs. Nursing turnover has emerged as a recent concern with a substantial influence on the financial efficiency and quality of healthcare worldwide. In the literature, turnover has been characterized as the “quantity of employees passing on an organization either for personal reasons because of some dissatisfactions in the working environment or being laid off by the employer because of specific issues looked at by the organization” and has been caused by job insecurity [10].

Age, commitment, gender, job security, profession, income, and hierarchical environment have all been determinants of turnover. Job satisfaction is likewise an indicator of turnover [11]. The yearly nursing turnover rates in Australia, Canada, and the United States are 15%, 20%, and 27%, respectively [12]. Recent nursing graduates and newcomers seem to have very high turnover rates. Staff-to-client ratios and healthcare results suffer accordingly. Studies have tried to distinguish predictors of nursing turnover and supply information to improve retention programs and reduce turnover rates. Among the recognized indicators are job dedication, satisfaction, anxiety, stress, and burnout [13]. Skills development and opportunities for advancement add to nursing job satisfaction and, accordingly, retention; subsequently, improving retention will be essential to achieving more self-sufficiency in the nursing labor force in Saudi Arabia.

Another study looked at expatriate nurses in Saudi Arabia employed on a year-to-year basis [14]. If their agreements are not broadened, it can prompt employee turnover, expanded mental stress, employment insecurity, and the threat of legal action, all of which affect patient safety and care quality. The presence of expatriate nurses can also give rise to significant issues related to their work and retention, particularly during times of distress. For instance, at the onset of COVID-19, some international nurses or other healthcare workers left for their home countries or faced difficulties returning to Saudi Arabia due to travel restrictions [15]. This would create an increased workload for Saudi workers, leading to unfortunate instances of their loss. Loyalty concerns among both non-Saudi and Saudi nurses further emphasize the need for an effective strategy that offers better professional development opportunities and supports job satisfaction for nurses in Saudi Arabia [16].

The gamble of the remaining nurses leaving or relocating to less upsetting work conditions increases accordingly. The significance of staff maintenance was underlined as one of the Vision 2030 objectives. However, the aforementioned objective has been fruitful in different areas, such as education, but not in Saudi healthcare, which is tormented by an ongoing lack of workers [17]. Addressing nurses’ intention to leave requires a comprehensive approach that takes into account the various factors that influence nurses’ job satisfaction and retention. By prioritizing the well-being and job satisfaction of nurses, healthcare organizations can help to ensure that they are able to provide high-quality care to patients.

Research has discovered that high salaries are connected to nurse retention, while low compensation is connected to a powerful urge to leave [18]. A coordinated assessment of earlier writing on nursing turnover in Saudi Arabia supported these findings, uncovering that month-to-month gross compensation was a driver of nurse turnover and expectation to leave [19]. Expatriate nurses are expected to pursue possibilities in other industrialized countries with better pay and working conditions. Therefore, hospital administrations ought to execute rules similar to those in other industrialized countries, which could result in enormous reserve funds and retention of the best healthcare personnel. The connection between the standard pay scheme and the desire to leave should be analyzed further to make suitable remuneration modifications.

International nurses outnumber Saudis in Saudi Arabia’s healthcare organizations. High turnover among them, as well as Saudi nurses, is a serious issue that needs to be tackled. The desire to leave the workplace is still a challenge in Saudi Arabia and probably around the globe. High turnover rates could impact hospitals’ quality of care and ways of selecting and training new employees. Identifying the elements that impact nurses’ intentions to leave can assist policymakers and stakeholders in keeping pace with the demands of the health system. To our knowledge, research in Saudi Arabia has not yet provided solutions for this issue. Therefore, this study aimed to determine important factors associated with nurses’ intentions to leave and what would bring them back. Retaining nurses is important as it helps to ensure continuity of patient care, reduces turnover costs, and improves job satisfaction and morale among nursing staff.

## 2. Materials and Methods

### 2.1. Study Design and Setting

This was a cross-sectional, multisite study of nurses in three hospitals in Saudi Arabia. The three hospitals were named A, B, and C for confidentiality. All hospitals were selected based on their easy accessibility. They are located in two different regions of Saudi Arabia. The size of their bed capacity and the diversity of medical conditions they treat were additional factors. Hospitals A and B are in the central city, with a total bed capacity of approximately 800 and 600 beds, respectively. Hospital C is located in the western region, with a total bed capacity of nearly 500 beds. Hospitals A and B are teaching hospitals, while Hospital C is a public hospital managed by the Ministry of Health. The three hospitals are recognized for providing high-quality healthcare services to patients. Different hospital environments may provide findings applicable to different health settings.

### 2.2. Sampling

This study was conducted on a convenience sample of nurses from the selected hospitals. Registered nurses with English language proficiency who work in one of the three hospitals were eligible to participate. English is the official language of nursing in Saudi Arabia; thus, we have focused exclusively on English speakers to avoid any language challenges that could hinder the accurate interpretation and analysis of data. We also aimed to provide reliable and easily understandable results in the official language of Saudi nursing. Nurses working for less than a year, lacking patient experience, or not interested in participating in the study were excluded. The G*power tool was used to estimate the required sample size, which provided a minimum of 143 to run multiple linear regression analyses. Missing data are common in research; thus, about 10% were added, providing a final minimum sample of 157.

### 2.3. Instrumentation

In addition to demographic variables, we developed a questionnaire to assess nurses’ job satisfaction and intention to leave work and return [20].

1. The job satisfaction scale consisted of 19 items measured on a 5-point Likert scale with responses as follows: strongly disagree = 1, disagree = 2, agree = 3, strongly agree = 4, it does not matter to me, or I don’t care much = 5, indicating high scores for high satisfaction level.
2. The intent to leave one’s current job consisted of 4 items measured on a 5-point Likert scale with responses as follows: most probably = 1, probably = 2, I don’t know/Not sure = 3, Unlikely = 4, Strongly Unlikely = 5, representing very unlikely leave for high scores.
3. The intention to return to nursing practice in Saudi Arabia included two descriptive items to assess the nurse’s desire to return to work and potential factors that could facilitate the return.

### 2.4. Data Collection and Analysis

Data were collected between September 2022 and April 2023. An anonymous questionnaire created on a secure online platform was used for data collection. The study link was sent to nurses at the three hospitals via unit managers. Interested and eligible participants were asked to read the consent forms prior to their participation. Upon their full understanding of the study procedure and agreement to participate, they could respond to the items. Additional recruitment strategies were employed to facilitate data collection, encompassing the utilization of social media platforms, word of mouth, and personal references. The aim of employing this diverse array of methods was to ensure an adequate number of participants for this study [21]. This would help improve the likelihood of obtaining a diverse and representative sample.

Collected data were analyzed using SPSS. The Kolmogorov–Smirnov test was conducted to assess the normality of the data distribution for the main variables [22]. The test was performed with a 95% confidence level, indicating that these data followed a normal distribution for both satisfaction and intent to leave. Missing data (less than 5% per item) were handled using the mean-value imputation for continuous variables [23]. No missing data were reported for categorical variables. To describe the sample and study variables, descriptive statistics and central tendency measures were run. Descriptive analyses were also used to indicate the reasons for returning to nursing if individuals had previously left the profession. An independent sample t-test and Pearson’s coefficient correlation test were run for bivariate analyses. Another bivariate analysis, one-way ANOVA, was also run to determine whether there were any statistically significant differences.

Finally, multiple linear regression was used to determine whether there was a relationship between satisfaction and intention to leave while controlling for demographic variables. In regression, we included all demographic variables that were theoretically relevant to job satisfaction and/or intention to leave or had reported relationships with these two variables in the current study or prior research studies. We controlled for demographic variables before presenting the results to mitigate any potential influence they may have had on the relationships with the main variables. Controlling these variables may also increase the likelihood of obtaining highly credible relationship outcomes. Further, these variables can provide important findings that might not be evident in bivariate analyses. Some demographics with several categories were collapsed into binary format for regression analyses. We also assessed the internal consistency of our structured scale. The significance level was set at 0.05.

## 3. Results

A total of 205 nurses completed the survey. The majority (*n* = 189) were female, non-Saudi (*n* = 159), married (*n* = 121), earned a monthly income of SR7000 or more (*n* = 110), held a bachelor’s degree (*n* = 161), and sometimes received incentives or other motivation (*n* = 92) (Table 1). Table 1 also shows the participant’s ages (in years) (M = 35.8, SD = 7.16, range: 23–59), years of experience (M = 12.19, SD = 6.44, range: 1–34), and years working at their current hospital (M = 7.88, SD = 5.31, range: 1–29). Both job satisfaction (M = 47.26, SD = 11.59, range: 19–76) and intent to leave a current job (M = 14.18, SD = 4.36, range: 4–20) were reported. Most nurses reported the main reason for returning to nursing if they left would be for better salary and benefits. Cronbach’s α of our structured scale was determined at 0.80.

Table 2 shows the mean differences between satisfaction, intention to leave, and some demographics. Using an independent sample t-test to analyze satisfaction, a significant difference was found only between the scores of nurses with different nationalities (Saudi vs. non-Saudi) (*p* < 0.05). Regarding the intent-to-leave variable, there was a significant difference reported between the scores of income levels (less than SR7000 vs. SR7000 or more) (*p* < 0.05). Other demographics showed no significant differences between satisfaction and intention to leave.

The Pearson’s coefficient correlation test demonstrated positive associations between age and years of experience in nursing (r = 0.872, *p* < 0.05) and between age and years working at the current hospital (r = 0.696, *p* < 0.05). Furthermore, the intention to leave was associated positively with years of experience (r = 0.152, *p* < 0.05) and negatively with nurse satisfaction (r = −0.551, *p* < 0.05). Further details are provided in Table 3.

One-way ANOVA was used to determine significant differences between nurse satisfaction, intention to leave, and some demographics (marital status, education level, number of children, hospitals worked in, incentives, and hospital type). Only incentives (*F* (2, 204) = 16.670, *p* < 0.001) and hospital type (*F* (2, 203) = 18.887, *p* < 0.001) were statistically different with nurse satisfaction. Further, marital status (*F* (3, 204) = 3.615, *p* = 0.014), education level (*F* (2, 204) = 5.864, *p* = 0.003), incentives (*F* (2, 204) = 8.872, *p* < 0.001) and hospital type (*F* (2, 203) = 10.452, *p* < 0.001) were all statistically different with nurses’ intentions to leave.

Table 4 and Table 5 show the regression analyses of satisfaction and intention to leave variables while controlling for demographic variables. Both satisfaction (*F* (13, 204) = 10.058, *p* < 0.001, R^2^ = 0.406) and intention to leave (*F* (13, 204) = 10.762, *p* < 0.001, R^2^ = 0.423) models were statistically significant. In Table 4, only income level (β = 0.159, *p* = 0.021), incentives (β = 0.186, *p* = 0.002), hospital type (β = 0.189, *p* = 0.005), and intention to leave (β = −0.454, *p* < 0.001) significantly influenced satisfaction. Table 5 shows gender (β = −0.122, *p* = 0.037), nationality (β = −0.210, *p* = 0.007), and satisfaction (β = −0.442, *p* < 0.001) to be significantly influencing intention to leave among participants. Years of experience and years working at the current hospitals were both not included in the regression analysis as it caused the coefficient estimates to become less precise and the overall model to lose precision.

## 4. Discussion

This study was conducted to determine important factors associated with nurses’ intentions to leave and what would bring them back. A significant association between nurses’ nationality and satisfaction level was reported. This finding is similar to prior research findings [24]. The authors reported that nurses of different nationalities experienced varying levels of job satisfaction, with some nationalities reporting higher satisfaction than others. Another study reported different findings [25]. This study was conducted in the Saudi context, and no significant association was found between nationality and overall job satisfaction. Our findings suggest that some factors, such as cultural background, language barriers, and social support networks, may impact nurses’ satisfaction levels based on their nationality [26].

Further, we found a significant association between nurse satisfaction and incentives; nurses receiving incentives are more likely to be satisfied with their nursing practice. Researchers examined the work environment and indicators of nurse satisfaction and reported that more incentives are required for nurses to improve their satisfaction [27]. Our findings are also similar to a previous research study in which giving sufficient incentives had a positive impact on nurse satisfaction [28]. The lack of satisfaction is related to work overload, perceived stress, and low payments. Incentives are one of the priorities that could motivate and attract staff, improving job satisfaction [29].

The results of the current study are similar to a previous study, which revealed that 9% of nurses intended to leave the nursing field because they were unsatisfied with their jobs [30]. The quality of treatment and patient outcomes would improve as a result of increased job satisfaction, which will also keep professionals from leaving their jobs [31]. To encourage retention, human resource policies should be created to meet the nurses’ needs while enhancing their quality of life and job satisfaction [32]. Our findings are also similar to the findings of other researchers who revealed that compared with nurses with work experience of 1 to 4 years, those with 20 years or more of experience were less likely to have the intention to leave (OR = 0.541, 95% CI = 0.347–0.844) [33]. Further, most nurses reported the main reasons for returning to nursing if they left it would be for better salary and benefits. An appropriate salary not only offers psychological and job stability to nurses but also influences their decision to return to a previous work environment if they encounter lower financial or competitive advantages elsewhere [34]. Adequate financial compensation, along with other benefits such as continuous professional development, plays a crucial role in enhancing the retention of valuable human resources for an extended period.

Many types of incentives could work for nurses, including financial incentives, flexible work schedules, education, overtime pay, tuition reimbursement, and recognition [35]. Incentives are appreciated by nurses and enhance their satisfaction. Nurse executives are encouraged to design cohesive work environments where rewards are given in appreciation for performance [36]. Further, incentives can be used to improve nurses’ retention and recruitment. Nurses, similar to any other professionals, expect to be paid fairly for the work they do. Offering a competitive salary and benefits package is essential for attracting and retaining nurses. Additionally, offering benefits such as good retirement plans can help nurses feel more secure and valued [37]. Further, a study by Buchan et al. (2018) found that competitive salary packages and comprehensive benefits were key factors influencing nurses’ decisions to re-enter the nursing workforce [38].

The intention to leave the workplace could be impacted by several factors, including payment. Low pay levels and poorly managed monthly payments have the potential to decrease nurse retention [34]. We found an association between nurses’ intentions to leave and income level (Table 3), and 46.3% of participants reported receiving less than SR7000 monthly (equivalent to US $1866). Our finding revealed that receiving low monthly income is considerably linked to nurses’ intentions to leave the workplace. Our finding is consistent with previous studies that reported low salary is inversely associated with nurse retention, whereas high salary induced a strong commitment to stay. Low monthly gross salary among staff nurses in Saudi Arabia is correlated with a high intention to leave [32].

Compared with other findings, the authors found that monthly salary was one of the significant predictive aspects of nurses’ intentions to leave their jobs [39]. Reviewing the literature on the relationship between nurses’ intentions to leave and income level showed that improving wages among nurses can reduce the intention to leave [32,40]. Hence, appropriate wages should be given to enhance nurse satisfaction and retention. It is important for healthcare organizations to offer competitive wages to attract and retain qualified nurses. Nurses are in high demand, and offering competitive wages can help healthcare organizations remain competitive in the job market and ensure that they are able to provide high-quality care to patients. It is also important to note that salary structures can vary across different hospitals and healthcare facilities in Saudi Arabia. Individual circumstances may also play a role in determining income levels for nurses.

Similar to bivariate analyses’ findings, the regression analysis reported that income level, incentives, hospital type, and intention to leave significantly influenced satisfaction among participants. Several studies have been found supporting such associations. For example, authors found that higher income levels were associated with increased job satisfaction [41]. Another study explored the influence of incentives on nurse satisfaction and reported a positive correlation between incentives and job performance [42]. Additionally, hospital type has been identified as a significant determinant of job satisfaction, indicating that nurses working in specialized hospitals reported higher levels of job satisfaction compared with those in general hospitals [39]. The intention to leave has also been linked to lower job satisfaction among nurses, as highlighted in the literature [39]. These findings underscore the importance of considering income, incentives, hospital type, and intention to leave in promoting nurse satisfaction and retention within healthcare organizations.

Further, gender and nationality have been identified as significant factors influencing the intention to leave the nursing profession. Research studies have shown that both gender and nationality play a role in shaping nurses’ decisions to leave nursing. For instance, female nurses were more likely to express an intention to leave their work than their male counterparts [43]. Similarly, nationality had a significant impact on nurses’ intention to leave, with nurses from certain nationalities exhibiting higher rates of intention to leave compared with others [32]. Such findings suggest that gender and nationality should be taken into consideration when implementing strategies to improve nurse retention and address the factors that contribute to their intention to leave the profession.

### 4.1. Study Limitations

The current study provides value by determining important factors associated with nurse satisfaction and the intention to leave, but some limitations should be noted. First, a cross-sectional design was used, which only considered associations between variables at a specific point in time. Second, convenience sampling was used in this study, which might lead to selection bias. Third, we limited our sample to include only fluent English speakers working at the three hospitals in Saudi Arabia, potentially reducing the generalizability of the results to other nurses who did not participate. Using self-reporting was another limitation with the disadvantage of response bias. Finally, the original developers of the questionnaire assessed its content validity but did not provide an explanation of the reliability measure [20]. Therefore, researchers should exercise caution when using this scale. Despite these limitations, we were able to provide valuable insights into nurses’ satisfaction and intention to leave the profession.

### 4.2. Study Implications

The findings from the current study suggest that providing incentives helps enhance nursing job satisfaction. The result may encourage nurse leaders to consider giving sufficient incentives to improve nurse satisfaction and care outcomes. To attain this, we recommend that nurse leaders and policymakers include nurses when designing and implementing incentive strategies. The major benefit of including nurses is to enhance nursing leaders’ awareness of the incentives that are appreciated by nurses and how to implement these incentives so that they can fulfill their intended purpose.

A key finding from this research study was the significant relationship between monthly salary and the intention to leave among nurses in Saudi Arabia. Nurses leaving their jobs is one of the main struggles that healthcare settings encounter. Therefore, we recommend that nursing policymakers in Saudi Arabia establish strategies to increase monthly salaries to compensate for workload and job dissatisfaction; for example, bonus pay such as overtime can help to overcome the issue of low monthly salaries. Such strategies will significantly impact the practice environment and help to increase job satisfaction and retain nursing staff.

Further, we found no significant differences between job status and satisfaction, though the satisfaction level between full-time and part-time employment can vary for several reasons. Full-time employees typically have a greater sense of job security and stability, as they often receive benefits such as health insurance and career growth. Part-time employees, on the other hand, often seek flexibility in their work schedule, allowing them to balance other commitments such as education, family, or personal interests. Therefore, individual preferences and circumstances that greatly impact job satisfaction should be investigated in future studies.

## 5. Conclusions

This study evaluated nurses in Saudi Arabia and important factors associated with satisfaction and the intention to leave their jobs. Our work indicated that job satisfaction and intention to leave are important factors affecting nurses’ enthusiasm. The present study determined that incentives had a positive impact on nurses’ satisfaction. The provision of incentives could be a reasonable factor in increasing the productivity of nurses and enhancing nursing care quality. In addition, our work indicated that monthly salary had a significant relationship with the intention to leave; a low monthly salary can affect job behavior and lead to disliking one’s career and wanting to leave. Future investigations into nurse satisfaction and intention to leave should determine what factors might improve monthly salaries and provide more incentives for nurses.

## Figures and Tables

**Table 1:** Sample characteristics (N = 205).

Characteristics	*n*	(%)
Age (Years) M = 35.8, SD = 7.16, Range: 23–59		
Gender		
Male	16	(7.8)
Female	189	(92.2)
Nationality		
Saudi	46	(22.4)
Non-Saudi	159	(77.6)
Marital status		
Single	79	(38.5)
Married	121	(59)
Divorced/Widow(er)	5	(2.5)
Number of children		
No children	95	(46.3)
One to five children	106	(51.7)
More than five children	4	(2.0)
Education level		
Diploma	30	(14.6)
Bachelor’s	161	(78.5)
Higher education	14	(6.8)
Level of monthly income		
Less than 7000SR	95	(46.3)
7000 or more	110	(53.7)
Years of experience (Years) M = 12.19, SD = 6.44, range: 1–34		
Years working at current hospital (Years) M = 7.88, SD = 5.31, range: 1–29		
Are you currently working as a bedside nurse?		
Yes	202	(98.5)
No	3	(1.5)
Job Status		
Permanent	112	(54.6)
Temporary (Contract)	93	(45.4)
Number of hospitals you have worked in		
One hospital	72	(35.1)
Two hospitals	68	(33.2)
Three hospitals	43	(21.0)
More than three hospitals	22	(10.7)
Received incentives or any motivation source		
Yes	27	(13.2)
Sometimes	92	(44.9)
No	86	(42.0)
Hospital type		
Hospital A	72	(35.1)
Hospital B	34	(16.6)
Hospital C	98	(47.8)

Note: M = Mean; SD = Standard Deviation; SR = Saudi Riyal.

**Table 2:** Mean differences between satisfaction, intention to leave, and some demographics.

Variable Mean Differences (*t*-Test)	Binary Categories	Satisfaction		*p*-Value	Intention to Leave		*p*-Value
		M	(SD)		M	(SD)	
Gender	Male	49.06	8.78	0.201	11.25	5.10	0.241
	Female	47.11	11.80		14.43	4.21	
Nationality	Saudi	52.50	8.25	0.001 *	10.84	3.90	0.449
	Non-Saudi	45.74	11.98		15.15	4.01	
Income level	Less than SR7000	48.41	11.45	0.659	14.63	3.52	0.001 *
	SR7000 or more	46.27	11.66		13.80	4.95	
Currently working?	Yes	47.13	11.62	0.097	14.26	4.33	0.489
	No	55.66	4.04		9.00	3.60	
Job Status	Permanent	46.36	11.40	0.839	14.12	4.54	0.162
	Temporary (Contract)	48.34	11.77		14.25	4.15	

* *p*-value < 0.05.

**Table 3:** Correlations between continuous variables.

	Age	Years of Experience	Years Working at the Current Hospital	Nurses Satisfaction	Intention to Leave
Age	1				
Years of experience	0.872 *	1			
Years working at the current hospital	0.696 *	0.690 *	1		
Nurses Satisfaction	−0.080	−0.081	0.035	1	
Intention to leave	0.105	0.152 *	0.045	−0.551 *	1

* *p*-value < 0.000.

**Table 4:** Multiple regression analysis for variables associated with satisfaction.

Variables	Unstandardized Coefficients		Standardized Coefficients	Sig.	Model Summary		
	B	Std. Error	Beta		R^2^	*p* Value	Adjusted R-Square
(Constant)	55.688	15.157		<0.001	0.406	0.000 *	0.366
Age	0.125	0.115	0.076	0.277			
Gender	−0.835	2.562	−0.019	0.745			
Nationality	2.322	2.186	0.084	0.290			
Marital status	0.829	1.895	0.035	0.662			
Education level	−2.345	2.734	−0.051	0.392			
Number of children	−0.945	1.913	−0.041	0.622			
Income level	−3.679	1.586	0.159	0.021 *			
Currently working as a bedside nurse	−1.239	5.749	−0.013	0.830			
Job status	0.107	1.440	0.005	0.941			
Number of Hospitals worked in	−0.343	1.430	−0.014	0.811			
Incentives	4.359	1.413	0.186	0.002 *			
Hospital type	4.384	1.559	0.189	0.005 *			
Intention to leave	−1.207	0.174	−0.454	<0.001 *			

* *p*-value < 0.05.

**Table 5:** Multiple regression analysis for variables associated with intention to leave.

Variables	Unstandardized Coefficients		Standardized Coefficients	Sig.	Model Summary		
	B	Std. Error	Beta		R^2^	*p* Value	Adjusted R-Square
(Constant)	21.571	5.608		<0.001	0.423	0.000 *	0.384
Age	−0.005	0.043	−0.008	0.906			
Gender	−1.973	0.940	−0.122	0.037 *			
Nationality	−2.192	0.798	−0.210	0.007 *			
Marital status	0.993	0.700	0.112	0.158			
Education level	−1.644	1.010	−0.095	0.105			
Number of children	0.841	0.708	0.096	0.236			
Income level	−0.026	0.597	−0.003	0.965			
Currently working as a bedside nurse	2.323	2.127	0.064	0.276			
Job status	0.152	0.534	0.017	0.776			
# of Hospitals worked in	0.451	0.530	0.049	0.396			
Incentives	−0.515	0.536	−0.058	0.338			
Hospital type	−0.210	0.590	−0.024	0.723			
Satisfaction	−0.166	0.024	−0.442	<0.001 *			

* *p*-value < 0.05.

## Data Availability

Data are not shared due to privacy and ethical restrictions.
